# Developing a MLC modifier program to improve fiducial detection for MV/kV imaging during hypofractionated prostate volumetric modulated arc therapy

**DOI:** 10.1002/acm2.12614

**Published:** 2019-05-22

**Authors:** Laura Happersett, Ping Wang, Pengpeng Zhang, James Mechalakos, Guang Li, Eleanor Eley, Michael Zelefsky, Gig Mageras, Antonio L. Damato, Margie Hunt

**Affiliations:** ^1^ Memorial Sloan Kettering Cancer Center New York NY USA

**Keywords:** fiducial tracking, image‐guided radiotherapy, motion management, MV/kV imaging, prostate

## Abstract

**Purpose:**

To develop an Eclipse plug‐in (MLC_MODIFIER) that automatically modifies control points to expose fiducials obscured by MLC during VMAT, thereby facilitating tracking using periodic MV/kV imaging.

**Method:**

Three‐dimensional fiducial tracking was performed during VMAT by pairing short‐arc (3°) MV digital tomosynthesis (DTS) images to triggered kV images. To evaluate MLC_MODIFIER efficacy, two cohorts of patients were considered. For first 12 patients, plans were manually edited to expose one fiducial marker. Next for 15 patients, plans were modified using MLC_MODIFIER script. MLC_MODIFIER evaluated MLC apertures at appropriate angles for marker visibility. Angles subtended by control points were compressed and low‐dose “imaging” control points were inserted and exposed one marker with 1 cm margin. Patient's images were retrospectively reviewed to determine rate of MV registration failures. Failure categories were poor DTS image quality, MLC blockage of fiducials, or unknown reasons. Dosimetric differences in rectum, bladder, and urethra D1 cc, PTV maximum dose, and PTV dose homogeneity (PTV HI) were evaluated. Statistical significance was evaluated using Fisher's exact and Student's *t* test.

**Result:**

Overall MV registration failures, failures due to poor image quality, MLC blockage, and unknown reasons were 33% versus 8.9% (*P* < 0.0001), 8% versus 6.4% (*P* < 0.05), 13.6% versus 0.1% (*P* < 0.0001), and 7.6% versus 2.4% (*P* < 0.0001) for manually edited and MLC_MODIFIER plans, respectively. PTV maximum and HI increased on average from unmodified plans by 2.1% and 0.3% (*P* < 0.004) and 22.0% and 3.3% (*P *< 0.004) for manually edited and MLC_MODIFIED plans, respectively. Changes in bladder, rectum, and urethra D1CC were similar for each method and less than 0.7%.

**Conclusion:**

Increasing fiducial visibility via an automated process comprised of angular compression of control points and insertion of additional “imaging” control points is feasible. Degradation of plan quality is minimal. Fiducial detection and registration success rates are significantly improved compared to manually edited apertures.

## 1 INTRODUCTION

Radiation techniques for the treatment of prostate cancer have dramatically changed over the past 25 years, from conventionally fractionated 3D conformal therapy to intensity‐modulated radiation therapy (IMRT) without image guidance to IMRT with image guidance (IGRT). Zelefsky et al^1^ determined that the use of IGRT, specifically the addition of image guidance with implanted fiducial markers for setup led to the reduction of urinary complications plus improved biomedical control for high‐risk patients. Furthermore, with the reduction of setup error, the prostate PTV margins could be reduced.^2^ With reduced margins and more accurate setup, hypo‐ and moderately hypo‐fractionated treatment regimens have become more prevalent. At our institution, an ultra‐hypofractionated regimen consisting of five fractions over 1–2 weeks is routinely used. In addition to the shortened treatment course compared to conventional fractionation, this approach also allows for dose escalation to the prostate. Because of the high dose per fraction, it is imperative to maintain a treatment program that reproducibly positions the patient day‐to‐day and monitors and corrects prostate motion during treatment. Implanted gold fiducial markers, as a prostate surrogate, can be used for both accurate patient setup and tracking. Image‐based systems which rely on single periodic kV images for monitoring are commercially available on standard linear accelerators. With such systems, shifts can be detected in two dimensions only and cannot provide full information about the dosimetrically important beams‐eye‐view direction. The addition of MV images, always orthogonal to the kV image, overcomes this limitation and such a technique, combining kV with short‐arc MV digital tomosynthesis (DTS) has been developed for IGRT during volumetric art therapy (VMAT).^3^, ^4^, ^5^, ^6^, ^7^

Unfortunately, fiducial marker visibility on the MV images can be an obstacle when monitoring prostate motion using synchronized MV/kV imaging due to blockage of the fiducials by the MLC. This article describes and evaluates two methods of modifying the VMAT beam to ensure exposure of at least one fiducial on the MV image with an imaging frequency sufficient for intra‐treatment motion monitoring of the prostate.

## 2 MATERIALS AND METHODS

Twenty‐Seven hypofractionated prostate patients with implanted fiducial markers treated between October 2014 and November 2017 were included in this study. They were all on an IRB approved study that retrospectively evaluated MV/kV data collected during routine radiotherapy. Fourteen patients received CT scans with MR fusion while 11 patients were treated using MR as the sole imaging method for planning. Prescription doses ranged from 37.5 to 45 Gy in 5 fractions, with majority of patients receiving 40 Gy. The average PTV D95% was 98 ± 1%.

Patients were planned with volumetric arc therapy (VMAT) on a commercial planning system (Eclipse V13.6, Varian Medical Systems, Palo Alto, CA). The plans consisted of two full arcs with control points every 2 degrees except in the beginning and end where 1 degree was used. One of the patient's plan had two additional arcs which were chosen by planner to improve conformality.

Patients were treated on linear accelerators equipped with MV and kV imaging (TrueBeam™, Varian Medical Systems, Palo Alto, CA). MV images were acquired continuously at a frequency of 10 Hz. kV images were acquired at set gantry intervals of 20 degrees, approximately 19 kV images for each arc, every 5–10 s. At the triggered kV gantry angles, the corresponding MV images were used to reconstruct short‐arc (3°) MV digital tomosynthesis (DTS) images. The VMAT plans were highly modulated and, on average, the fiducial markers were blocked 60–80% of the time in our patient population. To enable at least one marker visibility on the MV image, the plans were modified by one of two methods to expose one marker at the appropriate control points. These modifications did not violate the mechanical limitations of the TrueBeam linac and caused no beam interlocks or beam offs.

The first method, MLC manual modification, uses a feature in Eclipse where you can edit each leave position in beam properties. At control points where kV imaging was triggered, the MLC aperture was evaluated and if no fiducial markers were exposed, the MLC position was manually edited for three consecutive control points with margins between 0 and 5 mm. Margins variability was due to the nature of the technique which included human intervention and not a method where automatic margins were applied. These modifications were done without changing control point angles, dose rate or meterset weight. Modified plans were calculated in Eclipse. Enlarging MLC apertures inevitably increased the overall dose and plans were normalized to meet planning criteria. For some plans, the manual edit process had to be repeated because after the normalization of the first attempt, the plan coverage had unacceptably deteriorated. For these cases, the manual modification, plan recalculation, and normalization were repeated until an acceptable plan was obtained. There were 12 patients in this cohort.

**Figure 1:**
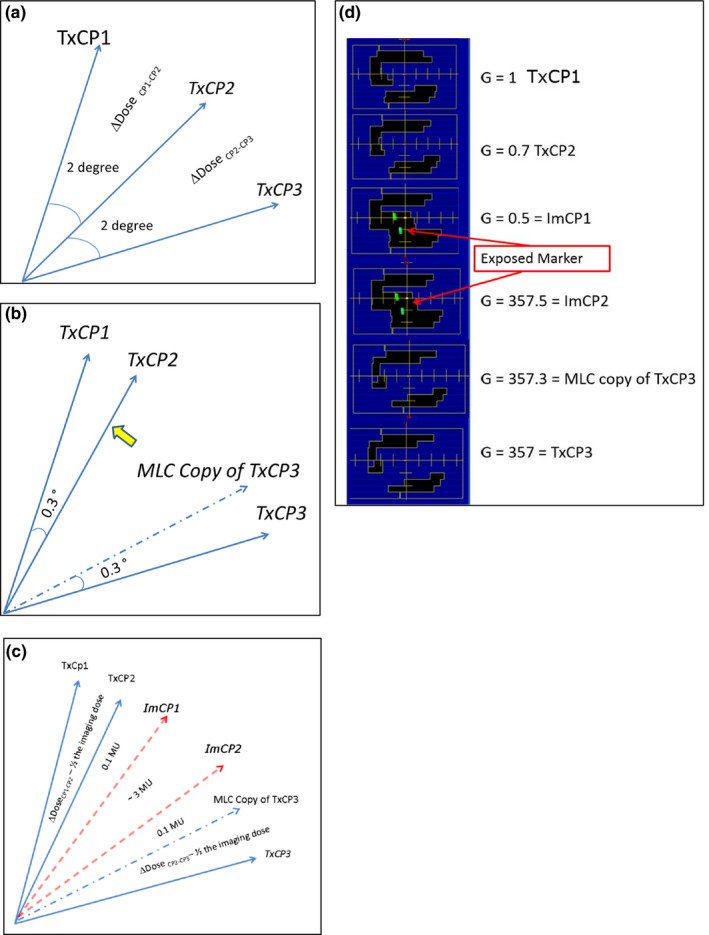
(a–d) outlines the MLC_MODIFIER design. (a) The original arc before any modifications are made. Three treatment control points (TxCP1,2,3) are separated by 2 degrees and the dose during each sub arc is displayed. (b) The MLC_MODIFIER program compresses the angles subtended by the treatment control points and adds an additional TxCP which has the same MLC positions of TxCP3. (c) Imaging control points (ImCP1, 2) are added and the dose of these imaging control points are subtracted from the treatment dose. (d) an example of the MLC positions is displayed for control points displayed in (d). The MLC_MODIFIER program, modifies the MLC position so the fiducial markers are exposed in ImCP1 and ImCP2.

The second approach modified the VMAT apertures via an Eclipse Scripting API plug‐in called MLC_MODIFIER. At appropriate angles, MLC_MODIFIER evaluates the fiducial marker exposure by measuring the distance between the edge of the fiducial contour and the MLC and action is taken if that distance is less than 1.5 cm. Figure 1(a)–1(d) describes the changes made to the arc with MLC_MODIFIER. First, the angles subtended by treatment control points were compressed. As illustrated in Fig. 1(b), the original angle between treatment control point 1 (TxCP1) and TxCP2 was reduced from 2° to 0.3°. Second, low‐dose, approximately 3 MU, “imaging” control points (ImCP1 and ImCP2) were inserted and dose delivered during the imaging arc segment was subtracted from treatment dose, as in Fig. 1(c). Between TxCPs and ImCPs, 0.1 MU is delivered to avoid disruptive beam hold. Finally, the imaging control points’ MLCs were modified to expose one fiducial with 1 cm margin as in Fig. 1(d). TxCP1, TxCP2, and TxCP3's MLC positions were not changed from the original plan. Overall, there are 68–91 out of total 356 control points involved in this modification. The modified plan was imported into Eclipse, dose calculated and normalized, if necessary. There were 15 patients in this cohort.

The MLC_MODIFIER method differs from the manual modification method in three ways. MLC_MODIFIER assigns appropriate imaging dose (~3 MUs) between imaging control points. This dose is then subtracted from the MUs delivered between treatment control points. The manual method does not alter the dose between control points, and therefore potentially gives greater dose to critical structures when the MLC aperture is modified. A second difference is the margin used for both methods. MLC_MODIFIER program automatically exposed the marker by 1 cm. The manual method was done by a physicist and the margin was variable. Finally, MLC_MODIFIER is an automatic script which takes approximately 10 unsupervised minutes to run, unlike the manual method which took one physicist at least 45–75 min to modify the plan.

In real time, a custom‐developed image registration program^3^, ^4^, ^5^ uses a fiducial marker template, the DTS MV images and the kV image to localize the fiducial marker(s) in addition to calculating and displaying the motion trace.

**Figure 2:**
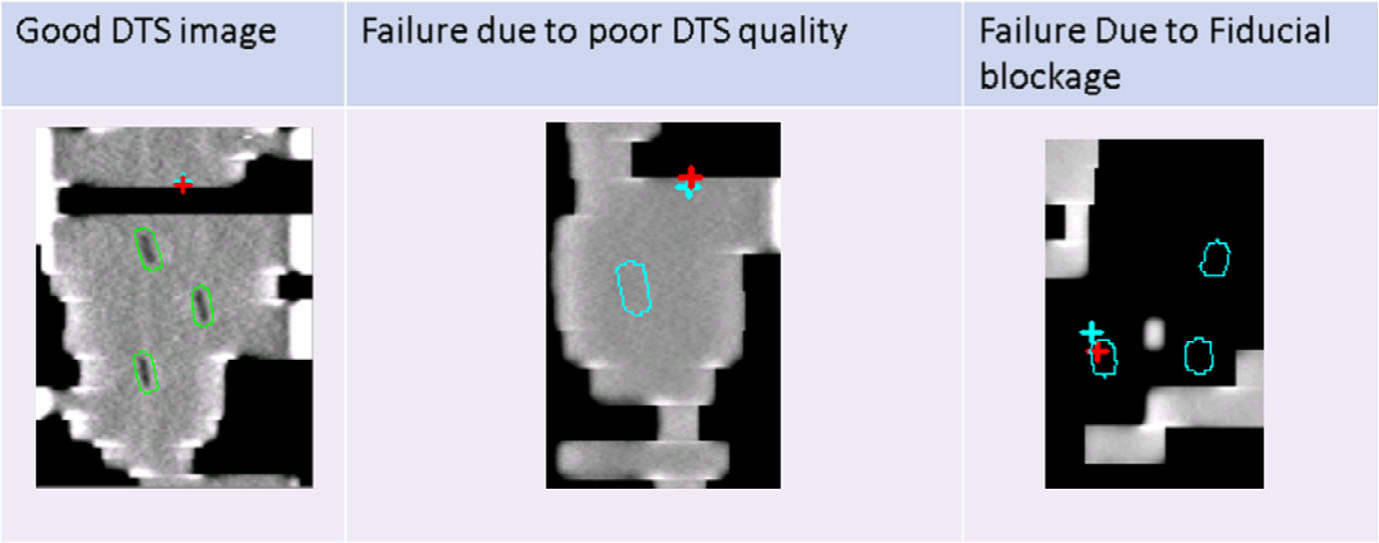
Examples of DTS images with and without registration failures. Fiducial contours are displayed in green when registration did not fail and in blue when the registration failed.

All 27 patients were treated with intra fraction monitoring using the described MV/kV regimen. Fiducial marker template and MV DTS registration results were evaluated retrospectively. Causes of registration failures were noted to be either poor DTS quality or MLC blockage. Example of these failures can be seen in Fig. 2. Other instances of registration failure without known cause were detected by noting the registration differences in the cranial caudal direction of 1 mm or greater. The frequency of each type of registration failures was evaluated for both methods. Statistical significance was evaluated using Fisher's exact test with a *P* values of less than 0.05 was considered significant.

Dosimetric differences between the modification methods were evaluated for both target and critical structures. Differences in rectum, bladder, and urethra D1CC was evaluated in addition to differences in the PTV maximal dose and dose homogeneity. PTV dose homogeneity (PTV HI) was defined as PTV HI = 100 × (D_maximum_–D_98%_)/PTV_mean_. Statistical significance was evaluated using Student's *t* test with *P* values of less than 0.05 was considered significant.

## 3 RESULTS

Data for 135 treatment sessions were evaluated. 1960 and 2376 registrations were evaluated for the manually modified and MLC_MODIFER cohorts, respectively.

The overall registration failure rates were significantly higher for the manually modified plans compared to the MLC_MODIFIER plans (33% vs 8.9% *P* < 0.0001). Failures for image quality were similar (8% vs 6.4% *P* < 0.05) but the failures due to blocked fiducials were significantly higher with the manually modified plans (13.6% vs 0.1% with *P* < 0.0001). Failures only detected by registration differences in the cranial caudal directions of more than 1 mm were higher in the manually modified plans compared to the MLC_MODIFIER plans (7.6 vs 2.4 % *P* < 0.0001).

There was a slightly greater amplitude in motion between the groups, 2.1 vs 1.3 mm for the manually modified and MLC_MODIFIER cohorts, respectively. For the patients in the manually modified cohort group whose motion amplitude that was greater than the average, the failure rates per patient was about 26% versus the group average of 33%. The increased motion amplitude did not predict an increased the failure rate.

The average beam on time difference for the two methods was −0.7% and −0.9% for the manually modified plans and MLC_MODIFIED plans, respectively, and was not found to be significant (*P* = 0.6).

Significant differences in the PTV maximum doses were observed between the original plan and the modified plan with the manually modified technique compared to MLC_MODIFIER. For the manually modified plans, the average maximum dose increased from 107.3 to 109.4%, on average 2 ± 1%, while for MLC_MODIFIER, the average maximum dose increased from 107.9% vs 108.3%, on average 0.3 ± 1% (*P* < 0.004). In addition, observed differences of PTV HI between the original plans and modified plans were significantly larger for the manually modified cohort compared to the MLC_MODIFIER, 22.0 ± 14.2% vs 3.3 ± 7.5%, respectively, with *P* < 0.004. Percent changes in rectum, bladder, and urethra D1CC were less than 0.7% and there were no significant differences between the methods.

## 4 DISCUSSION

The goals of introducing MLC_MODIFIER was to streamline the planning process which included reduction of human intervention and error, increase throughput and produce plans more consistent to the original plans. First, the reduction of time it takes to run MLC_MODIFIER compared to the manual method makes great strides to help increase throughput. The next version of MLC_MODIFIER, we hope to only take 2 or 3 min an even farther improvement.

In addition, to saving time, the registration results were much improved with MLC_MODIFIER method. Overall registration failures were far less for the MLC_MODIFIER plans compared to the manually edited plans. This is primarily due to the decrease in failures caused by blocked MLC and unknown reasons. In the MLC_MODIFIER schema, MLC positions are edited to expose one fiducial with a 1 cm margin while the manually modified plans had variable margins (0.0–5 mm). It could be hypothesized that if you increased the manual margin to 1 cm, you could obtain the same registration results. When we began with the manual approach, we tried larger margins, but found that plan dose would increase by 5–10% and renormalizing would cause an unacceptable degradation of PTV coverage. With the MLC_MODIFIER method, dose delivered via the low‐dose imaging control points is subtracted from the treatment control points and limits this effect, therefore enabling the use of larger margins. Because of this, the rate of registration failures due to MLC blockage dropped significantly from the manually edited plans to almost negligible for MLC_MODIFIER plans.

Further, it can be investigated, with MLC_MODIFIER if a balance in margin size and failure rate can be achieved. The ideal detection rate is 100% for localization of the prostate during treatment. Decreasing the margin around the marker could improve the plan dosimetric quality but could run a risk of compromising detection rate. In the future, we will investigate an optimal setting of the margin.

To tackle registration failures due to poor image quality, we propose the next version of MLC_MODIFIER vary the MU given at the imaging control based on patient separation. For example, instead of assigning 3 MU between imaging control points, the program would assign a value between 2 and 4 MU based on patient separation.

Finally, changes in plan quality were reduced with the MLC_MODIFIER plans as seen by smaller changes in PTV maximum and PTV HI. It is desirable for the modified plan to be as close in plan quality as the original plan. This way, there is less chance for either a replan or to have to re run the modification technique a second time.

In general, because MLC_MODIFIER had fewer failures, this lead to more accurate monitoring results during treatment. We have already treated over 230 patients using the MLC_MODIFIER method.

## 5 CONCLUSIONS

We have created an automated control point modification script that increases fiducial visibility in MV imaging without compromising plan quality. The fiducial detection rate during MV/kV imaging procedure is significantly improved as shown by the decreased rate of all three failure modes. Implementation of MLC_MODIFIER, as a plug‐in for Eclipse, is now being used routinely in the clinic.

## CONFLICT OF INTEREST

None.
